# Terminal complement complex deposition on chondrocytes promotes premature senescence in age- and trauma-related osteoarthritis

**DOI:** 10.3389/fimmu.2024.1470907

**Published:** 2025-01-14

**Authors:** Leonie Ruths, Jana Hengge, Graciosa Q. Teixeira, Melanie Haffner-Luntzer, Anita Ignatius, Jana Riegger

**Affiliations:** ^1^ Division for Biochemistry of Joint and Connective Tissue Diseases, Department of Orthopedics, Ulm University Medical Center, Ulm, Germany; ^2^ Institute of Orthopedic Research and Biomechanics, Ulm University Medical Center, Ulm, Germany

**Keywords:** osteoarthritis, complement system, terminal complement complex, senescence, interleukin-6

## Abstract

**Background:**

The complement system is locally activated after joint injuries and leads to the deposition of the terminal complement complex (TCC). Sublytic TCC deposition is associated with phenotypical alterations of human articular chondrocytes (hAC) and enhanced release of inflammatory cytokines. Chronic inflammation is a known driver of chondrosenescence in osteoarthritis (OA). Therefore, we investigated whether TCC deposition contributes to stress-induced premature senescence (SIPS) during aging *in vivo* and after *ex vivo* cartilage injury.

**Methods:**

Femoral condyles of male 13-week-old and 72-week-old CD59-ko (higher TCC deposition), C6-deficient (insufficient TCC formation), and C57BL/6 (WT) mice were collected to assess age-related OA. Furthermore, macroscopically intact human and porcine cartilage explants were traumatized and cultured with/without 30% human serum (HS) to activate the complement system. Explants were additionally treated with clusterin (CLU, TCC inhibitor), N-acetylcysteine (NAC, antioxidant), Sarilumab (IL-6 receptor inhibitor), STAT3-IN-1 (STAT3 inhibitor), or IL-1 receptor antagonist (IL-1RA) in order to investigate the consequences of TCC deposition. Gene and protein expression of senescence-associated markers such as CDKN1A and CDKN2A was determined.

**Results:**

In the murine aging model, CD59-ko mice developed after 72 weeks more severe OA compared to C6-deficient and WT mice. mRNA analysis revealed that the expression of Cdkn1a, Cdkn2a, Tp53, Il1b, and Il6 was significantly increased in the cartilage of CD59-ko mice. In human cartilage, trauma and subsequent stimulation with HS increased mRNA levels of CDKN1A, CDKN2A, and IL6, while inhibition of TCC formation by CLU reduced the expression. Antioxidative therapy with NAC had no anti-senescent effect. In porcine tissue, HS exposure and trauma had additive effects on the number of CDKN2A-positive cells, while Sarilumab, STAT-IN-1, and IL-1RA reduced CDKN2A expression by trend.

**Conclusion:**

Our results demonstrate that complement activation and consequent TCC deposition is associated with chondrosenescence in age-related and trauma-induced OA. We provided evidence that the SIPS-like phenotype is more likely induced by TCC-mediated cytokine release rather than oxidative stress. Overall, targeting TCC formation could be a future approach to attenuate OA progression.

## 1 Introduction

The musculoskeletal disease osteoarthritis (OA) is the primary cause of chronic pain and disability in the elderly worldwide. OA can be found in all synovial joints, but it is more pronounced in weight-bearing joints such as the knee and hip. Pathological changes in the knee joint are not limited to articular cartilage, as OA affects the whole joint, thus synovial inflammation, osteophyte formation, subchondral bone sclerosis, and degenerated ligaments are further hallmarks of OA (1, 2). The etiology of OA is considered multi-factorial with an interplay of systemic and local factors (e.g., aging, female gender, genetic predisposition, and overweight) (3). Local risk factors also include preceding traumatic injuries such as a torn meniscus or ligament, intra-articular fractures, and cartilage lesions (4). Pathomechanisms of primary OA and post-traumatic OA (PTOA) have been studied for decades, however, none of the current available treatments can reliably prevent OA progression (5, 6). Previous studies indicated that both the complement system and cellular senescence are involved in OA pathogenesis and specific targeting could be a future approach in OA therapy.

The complement system is an important part of the innate immune system and previous studies have indicated that it is crucially involved during OA and PTOA progression (7–11). Compared to healthy individuals, elevated levels of complement activation products, including C3a, C5b-9, C4d, and C3bBbP, were found in synovial fluid derived from OA patients and after an acute knee injury (12, 13). Besides the local expression by chondrocytes and synovial cells (10), intraarticular levels of complement components can also be influenced by bleedings due to knee injuries (11). Complement activation during OA progression is thought to be promoted by various micro-environmental changes (e.g., enhanced protease activity and accumulation of ROS) as well as damage-associated molecular patterns (DAMPs). The latter comprises cell- and matrix-derived components released during necrotic cell death and cartilage degradation (e.g., breakdown products of collagen type II) (2, 10, 14, 15). Activation of the complement system occurs in a cascade fashion which results in the generation of the anaphylatoxins C3a and C5a and formation of the terminal complement complex (TCC; also referred to as C5b-9). The TCC consists of C5b, C6, C7, C8, and multiple C9 molecules forming a pore in the cell membrane, thus inducing lysis and death of the target cells. However, sublytic TCC deposition is often observed in nucleated cells and associated with the secretion of proinflammatory cytokines (16) and mitochondrial dysfunction resulting in elevated reactive oxygen species (ROS) levels (17). In case of human articular chondrocytes (hAC), phenotypical alterations have been described as a consequence of TCC deposition (18). In contrast, functional loss of TCC due to C5- or C6-deficiency attenuated OA progression in injury-induced murine and rabbit OA models (8, 12). To circumvent TCC attack, cells including hAC express the membrane bound complement regulatory protein CD59, which prevents C9 polymerization (19, 20). Accordingly, CD59-deficiency resulted in enhanced cartilage degeneration in a murine PTOA model (12). Up to the present time, the exact role of the complement system and in particular TCC deposition during OA progression has not yet been fully unraveled.

Cellular senescence is described as a permanent cell cycle arrest characterized by the upregulation of cell cycle regulators such as p53 [encoded by tumor-suppressor protein 53 (*TP53*)], p16 [encoded by cyclin-dependent kinase inhibitor 2A (*CDKN2A*)], and p21 [encoded by (*CDKN1A*)] (21). Accumulation of senescent chondrocytes has been observed in articular cartilage of aged individuals and after joint injuries (22, 23). Furthermore, healthy mice were found to develop an OA-like phenotype after intraarticular injection of senescent fibroblasts into the knee joint (24). Selective elimination of senescent chondrocytes, in turn, was reported to reduce the risk of PTOA (23). The detrimental effect of senescent cells on surrounding tissues is most likely due to the senescence-associated secretory phenotype (SASP), which creates a pro-degenerative microenvironment (2, 24). The SASP secretome of hAC contains proinflammatory cytokines (e.g., IL-6, IL-8, and IL-1b) and proteases (e.g., MMP13) (25, 26). Inefficient clearance of senescent cells and subsequent persistence of cytokine release can promote senescence in neighboring cells in a paracrine manner, resulting in the accumulation of dysfunctional chondrocytes and cartilage degeneration (27, 28). Besides cytokines, enhanced levels of ROS are known to harm cells and thus cause so-called stress-induced premature senescence (SIPS) (29). Current therapeutic approaches against chondrosenescence target mainly senescence itself by removal of senescent cells or by suppressing their detrimental phenotype (30). Considering the hypocellularity of articular cartilage, identification and targeting of SIPS inducers might be more promising.

We propose that sublytic TCC deposition induces chondrosenescence either via subsequent inflammation or oxidative stress. Both are known inducers of senescence and are associated with sublytic TCC deposition (16, 17, 27–29). This hypothesis was investigated in an age-related *in vivo* and an *ex vivo* cartilage trauma model, respectively. We showed that complement activation and consequent TCC deposition was associated with a SIPS-like phenotype in chondrocytes. Inhibition of TCC formation resulted in a reduced expression of senescent associated genes. However, antioxidative therapy had no effect, while inhibition of IL-6 or IL-1β, in turn, reduced the expression of CDKN2A by trend.

## 2 Materials and methods

### 2.1 Animal care and mouse samples

Two different mouse models were used in this study. C6-deficient (C6-def) mice derived from mice with natural C6 mutations which were backcrossed from a C3H/He background (31) to a C57BL/6 background (32). Targeted deletions of exon 2 of the Cd59a gene resulted in the generation of the second mouse strain, CD59-knockout (CD59-ko) mice (33). Additionally, C57BL/6 wildtype (WT) mice were included. Animal experiments were carried out according to the European Union Directive 2010/63/EU and the international regulations for the care and use of laboratory animals (Animal Research: Reporting of *In Vivo* Experiments (ARRIVE) guidelines) were applied. The animal experiment was approved by the responsible local ethical committee (Animal Research Center of the Ulm University, Ulm, Germany; grant numbers, o.135-7 and o.135-12). Mice received standard mouse feed (ssniff R/M-H, V1535-300; Ssniff, Soest, Germany) and water ad libitum. Housing was performed in groups of up to five mice per cage with a 14-hour light, 10-hour dark rhythm. Since OA is more pronounced regarding cartilage degeneration in male mice (34–36) and that female mice have lower serum concentrations of some complement proteins, such as C6 and C9 (37), only male mice were used. As Wang et al. observed in mice after 72 weeks mild OA, mice were sacrificed and weight (**Supplementary Table 1**) at an age of either 13 weeks or 72 weeks (12). The condyles of the left hind leg were used for gene expression analysis. The right hind leg was used for histology and for this purpose fixated in formalin and subsequently decalcified with 20% EDTA (Sigma-Aldrich, Darmstadt, Germany) for 2 weeks.

### 2.2 Human samples

Samples were obtained from donors undergoing surgical knee replacement. Patients gave their written informed consent according to the terms of the Ethics Committee of the University of Ulm following the instructions of the Declaration of Helsinki (ethical approval number 353/18). Macroscopically intact cartilage tissue (Osteoarthritis Research Society International (OARSI) grade ≤ 1) (38) derived from human femoral condyles was used either for isolation of hAC, extraction of full-thickness cartilage explants, or immediately stored in 4% formalin for histology. Additionally, highly degenerated cartilage tissue (OARSI grade ≥ 3) was collected and processed for histology. Exemplary images of macroscopically intact cartilage tissue (OARSI grade ≤ 1) and highly degenerated cartilage tissue (OARSI grade ≥ 3) are provided in Riegger et al., 2023 and Ruths et al., 2024 (7, 39).

### 2.3 Isolation and cultivation of human articular chondrocytes

To isolate hAC, macroscopically intact cartilage was cut into small pieces. Tissue was digested with 0.2% pronase (Sigma-Aldrich) for 45 min and afterwards with 0.025% collagenase (Sigma-Aldrich) overnight at 37°C. Undigested pieces were removed by means of a 40 µm cell strainer. Cells were cultured in basal medium [1:1 DMEM (Live Technologies, Paisley, UK) and Ham´s F12 (PAN Biotech, Aidenbach, Germany), 10% fetal bovine serum (PAN Biotech), 1 g/L glucose, 0.5% L-glutamine (PAN Biotech), 0.5% penicillin/streptomycin (PAN Biotech), 10 μg/mL 2-Phospho-L-ascorbic acid trisodium salt (Sigma-Aldrich)] and split at a confluency of 80% and were used in passage 1 or 2. Cultivation was performed at 37°C, 5% CO2, and 95% humidity.

### 2.4 Preparation, cultivation and traumatization of human cartilage explants

Full-thickness cartilage explants (Ø 6 mm) were extracted from macroscopically intact cartilage. After isolation, explants were cultured at least 24 h in serum free medium [SFM: DMEM, 1 g/L glucose, 1% pyruvate (Sigma-Aldrich), 1% non-essential amino acids (Bio-Sell, Feucht, Germany), 0.5% L-glutamine, 0.5% penicillin/streptomycin, 10 μg/mL 2-Phospho-L-ascorbic acid trisodium salt, 0.1% Insulin-Transferrin-Selenium (Live Technologies)]. During running experiments, explants were stimulated for 4 days with 30% pooled human serum (HS; Innovative Research, Peary Court, USA), 20 μg/mL cartilage homogenate (HG), 2 mM N-acetyl cysteine (NAC; Sigma-Aldrich), and/or 30 μg/mL clusterin (CLU; R&D Systems, Wiesbaden, Germany).

Depending on the experimental set-up, cartilage explants were traumatized with a single impact (0.59 J) with an established drop-tower model prior to stimulation (40, 41). This allows to study trauma-induced alterations of cartilage tissue and hAC.

### 2.5 Preparation, cultivation and traumatization of porcine samples

Right hind legs of 6-month-old pigs were obtained from a local butcher. Cartilage explants (Ø 6 mm) were extracted from the medial femoral condyle and cultured in SFM_high_ containing 4.5 g/L glucose. Porcine cartilage explants were traumatized with a reduced impact energy of 0.47 J and stimulated with 30% HS or 30% heat inactivated (Hi) HS in SFM_high_. As we were interested in consequences of sublytic TCC deposition and HS was used for the porcine tissue, the incubation time was reduced and no HG was added. After 24 h, medium was replaced with fresh SFM_high_ supplemented with either 5 μg/mL Sarilumab (IL-6 receptor inhibitor; Selleck Chemicals, Houston, USA), 5 μM STAT3-IN-1 (STAT3 inhibitor; Selleck Chemicals) or 50 ng/mL IL-1RA (IL-1 receptor antagonist; PeproTech, Cranbury, USA). At day 4, a Live/Dead staining was performed. Remaining cartilage pieces were stored in 4% formalin for histological analysis.

### 2.6 Live/dead viability/cytotoxicity assay

A Live/Dead Viability/Cytotoxicity kit (Live Technologies) was used to investigate the percentage of living cells in cartilage explants. As previously described (40), a tissue section (0.5 mm thickness) was cut out of the unfixed explants and incubated for 40 min in a staining solution (1 µM calcein, 2 µM ethidium homodimer-1). Microscopical analysis was performed using a z-stack model (AxioVision software) and an Axioskop 2 mot plus microscope (Carl Zeiss, Oberkochen, Germany).

### 2.7 siRNA-mediated silencing of CD59

CD59 expression was knocked down in hAC by means of silencer select pre-designed siRNA (s2696, Live Technologies). Scrambled siRNA (Live Technologies) was used as negative control (siNC). The first knockdown was carried out in suspension. hAC were digested with a solution containing 1 mg/mL collagenase, 1 mg/mL protease, and 4 U/mL hyaluronidase (Sigma-Aldrich) for 1 h at 37°C. Next, hAC were seeded at a density of 24,000 cells/cm^2^ in 1 mL Opti-MEM (Fisher Scientific, Schwerte, Germany) and the transfection mix [Opti-MEM containing 1.5% Lipofectamine 3000 (Fisher Scientific) and 1% siRNA (10 μM)] was added. After an incubation of 4 h in the cell incubator, Opti-MEM was removed and basal medium was added. After 72 h, cells were trypsinized and seeded at a density of 21,000 cells/cm^2^. On the next day, the second knockdown was carried out with adherent cells. 3 days after the second transfection, cells were used for gene expression analysis and CD59-immunofluorescence (IF) to confirm the knockdown on mRNA and protein level. Additionally, hAC were incubated for 2 h with 30% HS, followed by a TCC-IF to confirm the knockdown on a functional level.

### 2.8 CD59- and TCC-IF

For the CD59-IF, cells were at first washed once with PBS and then protein block (Dako, Hamburg, Germany) was added for 60 min at 37°C. Next, a PE-labeled anti-human CD59 antibody (1:1200, B304708, Biozym, Hessisch Oldendorf, Germany) was incubated for 2 h. Unbound antibodies were removed by washing with PBS and cells were fixated with formalin (15 min). Nuclei were stained with Dapi (0.25 μg/mL, 10 min, Sigma-Aldrich).

In case of TCC-IF, the primary antibody (1:4000, ab55811, abcam, Cambridge, UK) was added for 2 h. Afterwards, a biotinylated goat anti-polyvalent solution (Dako) was incubated for 30 min, followed by the addition of an iFluor 568-straptavidin conjugate (1:100, ABD-16960, AAT Bioquest, Pleasanton, USA) for 20 min. Nuclei were stained with Dapi.

CD59 (red), TCC (red), and nuclei (blue) were visualized with an Axioskop 2 mot plus microscope (Zeiss, Oberkochen, Germany). The corrected total cell fluorescence (CTCF) was determined with ImageJ 2.9.0.

### 2.9 Gene expression analysis

Total RNA was extracted from cryopreserved cartilage explants or condyles of left hind legs from mice. A microdismembrater S (B. Braun Biotech, Melsungen, Germany) was used to disintegrate the tissue and RNA was isolated by means of the RNeasy Lipid Tissue Mini Kit (Qiagen, Hilden, USA). To isolate RNA of cultured cells, the RNeasy Mini Kit (Qiagen) was applied. All kits were carried out following the manufacturer’s instructions, including the Omniscript RT Kit (Qiagen) which was used for reverse transcription.

Quantitative real-time polymerase chain reaction (StepOnePlus Real-Time PCR System, Applied Biosystems) was used together with TaqMan Gene Expression Mastermix (Applied Biosystems) and Assays (**Supplementary Table 2**) to analyze gene expression. Self-designed primers were used in combination with SYBR Green PCR Mastermix (Applied Biosystems). *GAPDH* and *HPRT1* served as reference genes in mice and human samples. The ΔΔCt method was applied to calculate mRNA expression levels compared to the reference samples (either macroscopically intact cartilage tissue, untreated controls, or the median of 13-week-old WT mice).

### 2.10 Safranin O staining and histopathological assessment of OA grade

Paraffin embedded knee joints were cut, dewaxed, and rehydrated prior to staining. Sections were then incubated with Weigert´s iron hematoxylin (Merck, Darmstadt, Germany), then in 0.03% Fast Green (Sigma-Aldrich), followed by 0.1% Safranin O (Chroma, Köngen, Germany). Stained sections were analyzed with an Axioskop 2 mot plus microscope. OA grade was determined on the medial condyle by analyzing the osteoarthritis damage and size and maturity of osteophytes (42).

### 2.11 Immunohistochemistry and IF staining of murine knee joints

Paraffin embedded knee joints were cut, dewaxed and rehydrated prior to staining. CDKN1A was stained with an IHC staining protocol, while CDKN2A was stained by IF. Antigen retrieval was done by incubating the sections in 10 mM citrate buffer at 95°C for 20 min. In case of IHC, endogenous peroxidase was blocked with 3% methanol for 15 min. Sections were blocked with 10% goat serum for 30 min at 37°C. Staining was performed with either CDKN1A (1:50, MA5-14949, Live Technologies) or CDKN2A antibody (1:100, ab189034, Abcam). Secondary antibodies were either goat anti-rabbit biotinylated or AF594 conjugated (1:100, Invitrogen, Cat # A-11012). IF sections were counterstained with 1:1000 Hoechst dye.

### 2.12 Histology of cartilage explants

Paraffin embedded cartilage explants were cut, dewaxed, and rehydrated prior to staining. For immunohistochemistry (IHC) and IF, antigen retrieval was preformed depending on the used antibody. For TCC (1:250, A239, Quidel Ortho, San Diego, USA) sections were digested with 2 mg/mL hyaluronidase in 10 mM citrate buffer (30 min, 37°C). In case of CDKN1A (1:50, MA5-14949, Live Technologies), CDKN2A (1:200, ab108349, Abcam), and p53 (1:100, LS-B7723, LSBio, Shirley, USA) antigens were demasked with 10 mM citrate buffer (16 h, 65°C). Afterwards, primary antibodies were incubated overnight (4°C), followed by an incubation in 3% hydrogen peroxide (30 min). Subsequent IHC staining was performed with the LSAB2 System horseradish peroxidase kit (Dako, Glostrup, Denmark) and nuclei were stained with gill´s haematoxylin (Merck). In the case of IF, an iFluor 568-streptavidin conjugate (0.25 µg/mL, 16960, AAT Bioquest, Sunnyvale, USA) and DAPI (0.25 µg/mL, 10 min) was used.

Stained sections were analyzed with an Axioskop 2 mot plus microscope. At least 3 images of different sections and locations were quantified by manual counting to obtain the percentage of positive cells.

### 2.13 Statistical analysis

At least three independent experiments were carried out with cells or cartilage explants derived from different donors (biological replicates). Results are presented as box and whiskers with all data points and GraphPad Prism Version 10 was used for statistical analysis. Normally distributed data (Shapiro-Wilk test) was analyzed with an unpaired t-test, one-way ANOVA, or Pearson correlation. Unpaired Mann-Whitney test was chosen for not normally distributed data. Corrections of multiple comparisons were selected based on the recommendations of GraphPad Prism and are included in the figure legends. Significance level was set to p ≤ 0.05.

## 3 Results

### 3.1 CD59-ko results in increased expression of senescence markers and OA development in aged mice

**Figure 1:**
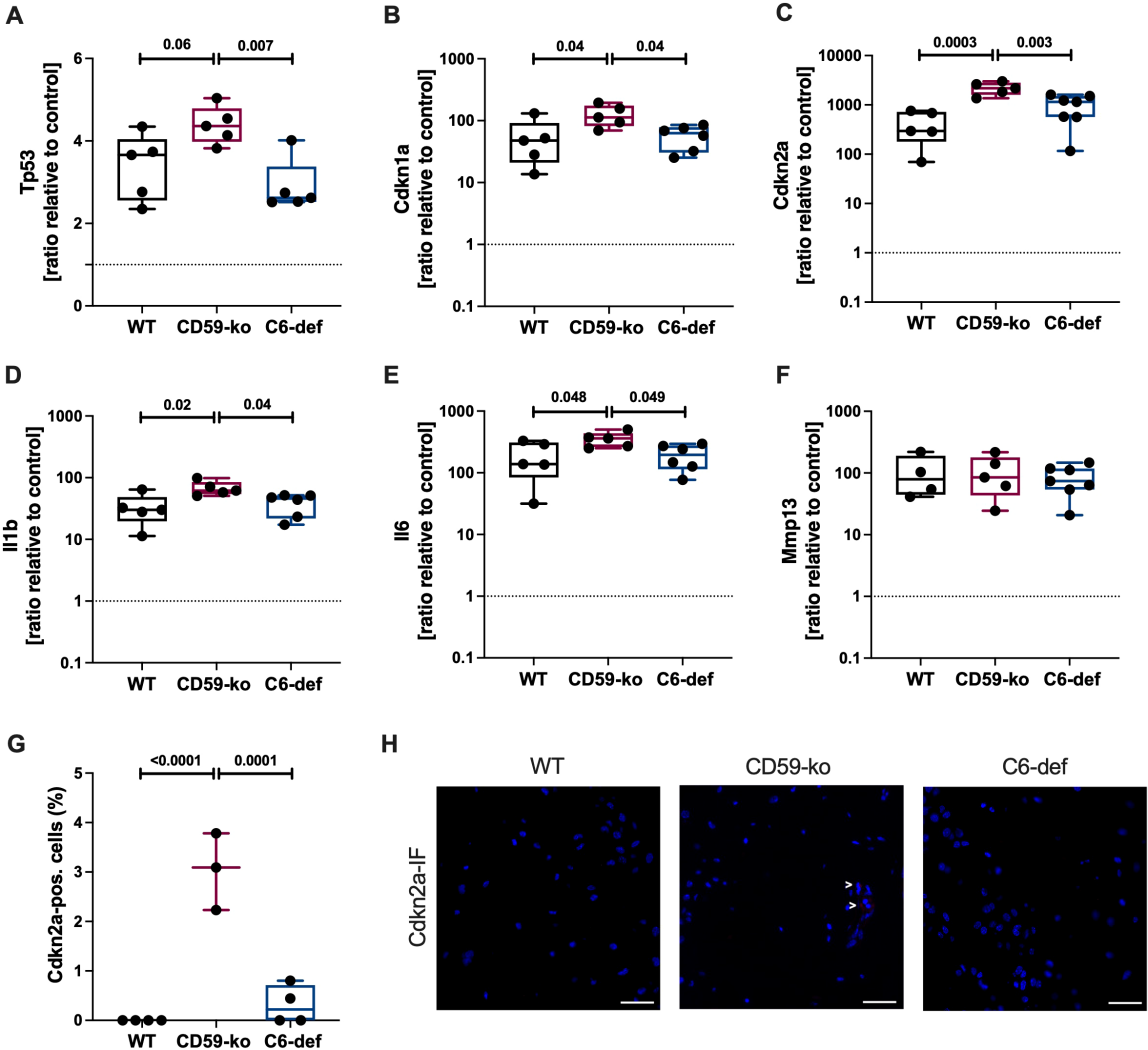
Gene and protein expression in femoral condyles of 72-weeks-old WT, CD59-ko, and C6-def mice. mRNA levels of **(A)** *Tp53*, **(B)** *Cdkn1a*, **(C)** *Cdkn2a*, **(D)** *Il1b*, **(E)** *Il6*, and **(F)** *Mmp13* were normalized to the median of 13-week-old WT mice; n ≥ 4. **(G)** Percentage of Cdkn2a-positive cells; n ≥ 3. **(H)** Representative images of Cdkn2a-IF. Scale bars equal 50 μm. Statistical analysis: one-way ANOVA with **(A, C)** Bonferroni´s, **(B, D, G)** Tukey´s, or **(E)** Šidák´s correction.

A murine aging model was used to investigate if the lack of TCC-specific complement regulator CD59 (CD59-ko mice) or insufficient TCC formation (C6-def mice) alters the expression of senescence-associated genes in the femoral condyles of 72-week-old mice. Overall, all tested genes were upregulated compared to 13-week-old WT mice (**Figures 1A–F**). The cell cycle regulators *Tp53*, *Cdkn1a*, and *Cdkn2a* were upregulated in CD59-ko mice compared to WT and C6-def mice (**Figures 1A–C**). Similar results were found for the mRNA expression of the SASP factors *Il1b* and *Il6* (**Figures 1D, E**). Both genes were higher expressed in CD59-ko mice. mRNA levels of *Mmp13* were not affected (**Figure 1F**). In the case of Cdkn2a, the upregulation was confirmed on protein level (**Figures 1G, H**). However, Cdkn1a expression was negative in all mice (**Supplementary Figure 1**). In addition, correlation of *Cdkn1a* and *Cdkn2a* with the SASP factors indicated that *Il1b* and *Il6* are the potentially relevant SASP factors in this OA model (**Supplementary Figure 2**).

**Figure 2:**
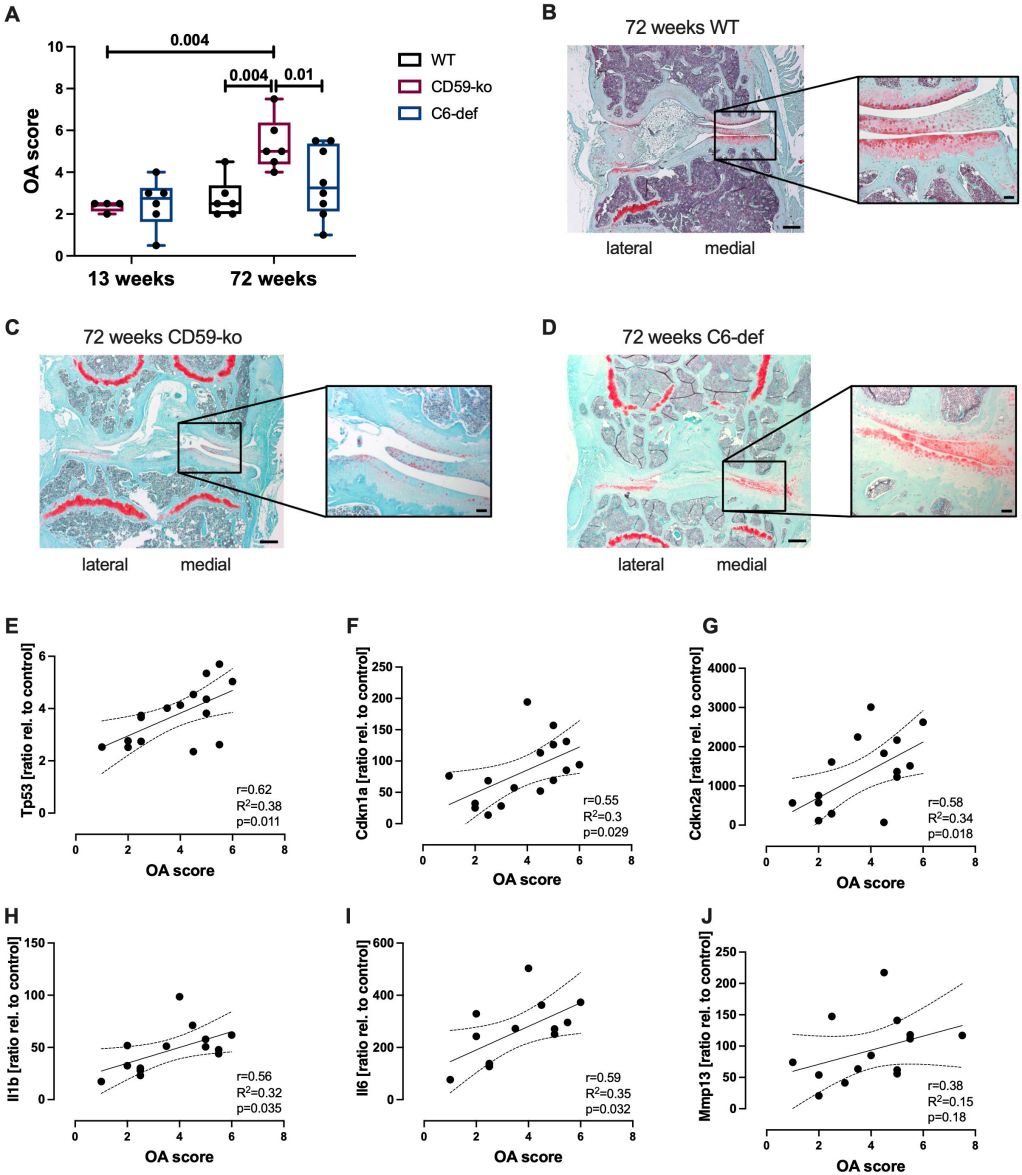
Histological evaluation of knee joints of 13-weeks and 72-weeks-old WT, CD59-ko, and C6-def mice. **(A)** OA Score of medial condyles, assessed by means of Safranin O staining; n ≥ 4. Exemplary images of Safranin O-stained knee joints of 72-week-old **(B)** WT, **(C)** CD59-ko, and **(D)** C6-def mice. Scale bars represent 200 μm and in magnification 50 μm. Correlation analysis of OA score and gene expression of **(E)** *Tp53*, **(F)** *Cdkn1a*, **(G)** *Cdkn2a*, **(H)** *Il1b*, **(I)** *Il6*, and **(J)** *Mmp13* of 72-week-old mice; n ≥ 13. Statistical analysis: **(A)** one-way ANOVA with Holm-Šidák´s correction; **(E–J)** Pearson correlation analysis with 95% confidence intervals.

Furthermore, OA score of young (13-week-old) and aged (72-week-old) WT, CD59-ko, and C6-def mice was determined based on Safranin O-stained sections (**Figures 2A–D**). In 13-week-old CD59-ko and C6-def mice no histomorphologic alterations were observed. Thus, the knee joint and cartilage tissue did not seem to be affected by the genetical alterations. However, in 72-week-old mice, clear differences between the groups were observed in comparison to CD59-ko mice. The OA score of CD59-ko mice was significantly higher than that of WT and C6-def mice. Loss of Safranin O staining intensity and formation of osteophytes was mainly observed in aged CD59-ko mice (**Figure 2C**). Additionally, the OA score was positively associated with the expression levels of *Tp53*, *Cdkn1a*, *Cdkn2a*, *Il1b*, and *Il6* (**Figures 2E–I**). A positive trend was found in the case of *Mmp13* (**Figure 2J**).

Overall, these results indicate that enhanced TCC deposition might be associated with OA development and chondrosenescence in aged mice.

### 3.2 CD59 knockdown facilitates enhanced TCC deposition in hAC

**Figure 3:**
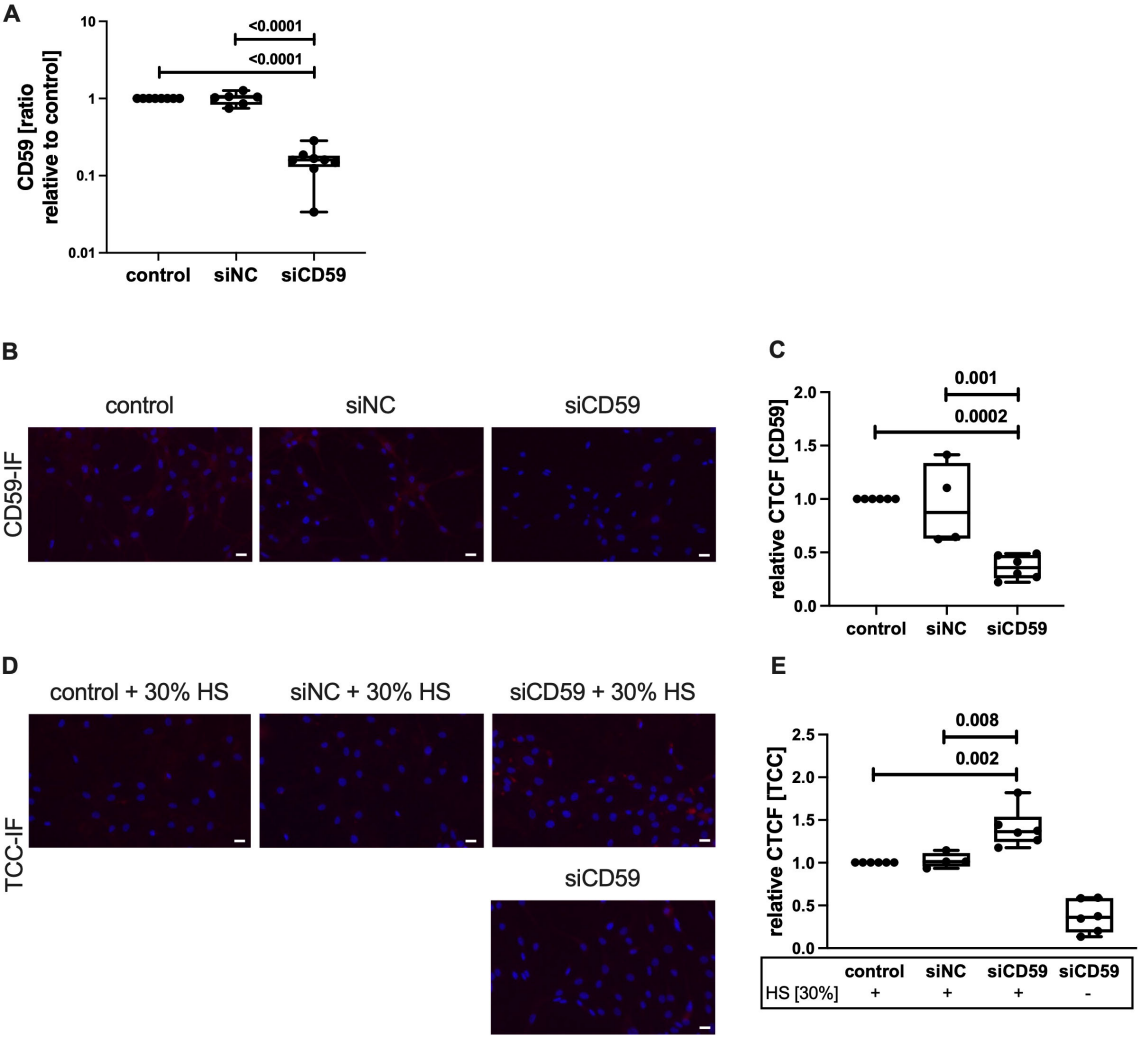
Knockdown of CD59 in hAC. hAC were transfected in suspension with either scrambled siRNA (siNC) or siRNA targeting CD59 (siCD59). On day 4, a second knockdown was carried out in suspension. **(A)** Knockdown was evaluated at day 7 on mRNA level. Gene expression was normalized to untransfected control; n ≥ 6. **(B)** Visualization of CD59-IF. Scale bar equals 20 μm. **(C)** Quantification of CD59 protein expression at day 7, relative to non-transfected control; n ≥ 4. **(D)** Visualization of TCC-IF. Scale bars equal 20 μm. **(E)** Quantification of TCC deposition at day 7 after exposition with 30% human serum (HS), relative to non-transfected control; n ≥ 4. Statistical analysis: **(A, C, E)** one-way ANOVA with Tukey´s correction.

To confirm that the lack of CD59 indeed leads to an enhanced TCC deposition upon complement activation, we performed an siRNA-mediated knockdown of CD59 in hAC. Knockdown was confirmed on mRNA level after 7 days (**Figure 3A**). *CD59* expression was significantly reduced, compared to the non-transfected control and hAC transfected with scrambled siRNA (siNC). Furthermore, IF staining of CD59 confirmed a successful knockdown on protein level (**Figures 3B, C**). After HS exposition for 2 h to activate the complement system, silencing of CD59 resulted in an increased TCC deposition on hAC (+36,4%) as compared to control cells (**Figures 3D, E**).

### 3.3 TCC deposition and p53 expression are increased in highly degenerated cartilage tissue

**Figure 4:**
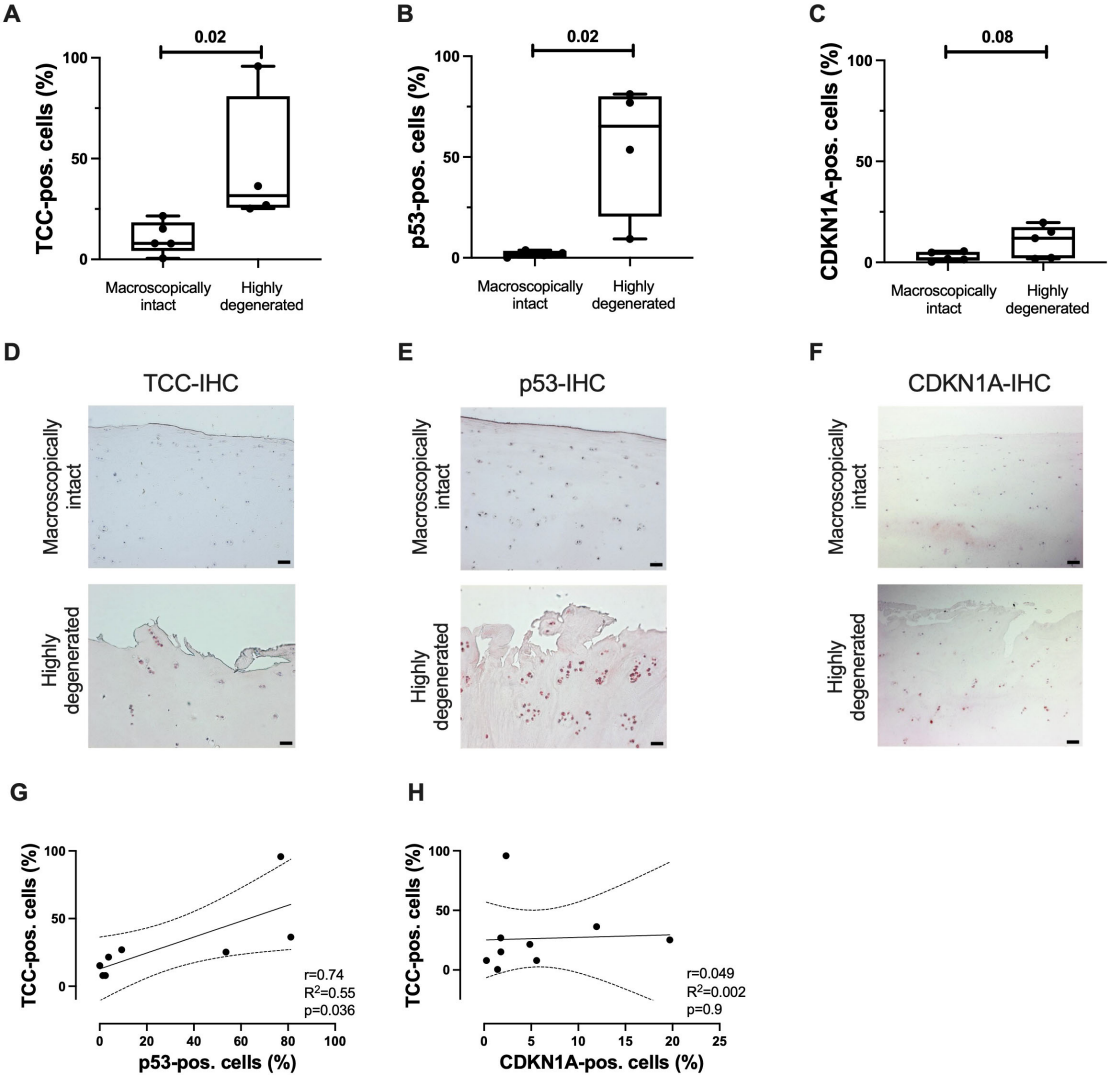
TCC deposition and expression of p53 and CDKN1A in human articular cartilage. Percentage of **(A)** TCC-, **(B)** p53-, and **(C)** CDKN1A-positve cells in highly degenerated (OARSI grade ≤ 1) and macroscopically intact tissue (OARSI grade ≥ 3); n ≥ 4. Representative images of **(D)** TCC-, **(E)** p53-, and **(F)** CDKN1A-IHC of articular cartilage. Scale bars equal 50 μm. Correlation analysis of number of **(G)** TCC- and p53-positive cells and **(H)** TCC- and CDKN1A-positve cells; n ≥ 8. Statistical analysis: **(A)** unpaired Mann-Whitney test; **(B, C)** unpaired two-tailed t-test; **(G–I)** Pearson correlation analysis with 95% confidence intervals.

Next, TCC deposition and presence of senescence-associated cell cycle regulators were analyzed in clinical samples of macroscopically intact and highly degenerated human articular cartilage. Significant more cells were TCC- or p53-positive in highly degenerated cartilage as compared to macroscopically intact tissue (**Figures 4A, B, D, E**). For CDKN1A, only a trend was observed (**Figures 4C, F**). Additionally, a positive association between the number of TCC- and p53-positive cells was found (**Figure 4G**). CDKN1A expression did not correlate with TCC deposition (**Figure 4H**). No association between the age of the donors and the percentage of positive cells was detected (**Supplementary Figure 3**).

### 3.4 Inhibition of TCC formation reduces expression of senescence-associated genes

**Figure 5:**
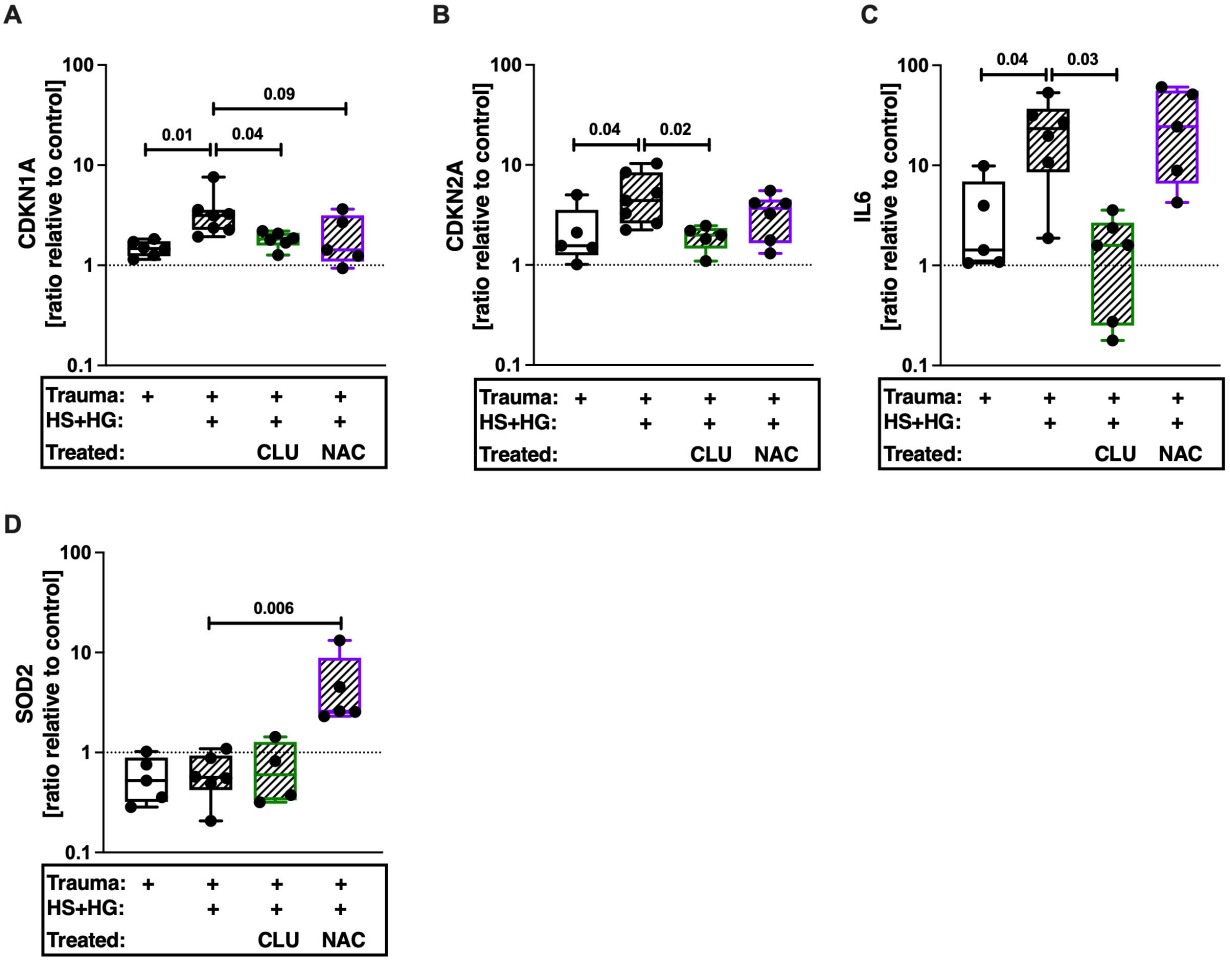
Gene expression of human articular cartilage. Traumatized cartilage explants were cultured for 4 days in presence of 30% human serum (HS) and 20 μg/mL cartilage homogenate (HG). Additionally, explants were treated with 30 μg/mL CLU or 2mM NAC. Gene expression of **(A)** *CDKN1A*, **(B)** *CDKN2A*, **(C)** *IL6*, and **(D)** *SOD2* was assessed. Unimpacted cartilage served as control; n ≥ 4. Statistical analysis: one-way ANOVA with **(A, D)** Šidák´s, **(B)** Dunnett´s, or **(C)** Holm-Šidák´s correction.

By means of a human *ex vivo* cartilage trauma-model, we wanted to determine whether TCC deposition induces the expression of senescence-associated genes. Traumatized cartilage explants were therefore treated with 30% HS and 20 μg/mL HG for 4 days. We previously showed that this combination leads to a high TCC deposition (18). Furthermore, this treatment induced gene expression of *CDKN1A*, *CDKN2A*, and *IL6* (**Figures 5A–C**). Inhibition of TCC formation by CLU in turn reduced expression of these genes. Antioxidative therapy by means of NAC did not affect the respective mRNA levels. Expression of *SOD2*, which protects cells from oxidative stress (43), was only upregulated in presences of NAC (**Figure 5D**).

### 3.5 Inhibition of inflammatory cytokines IL-6 and IL-1b reduces CDKN2A expression after trauma and HS exposition

**Figure 6:**
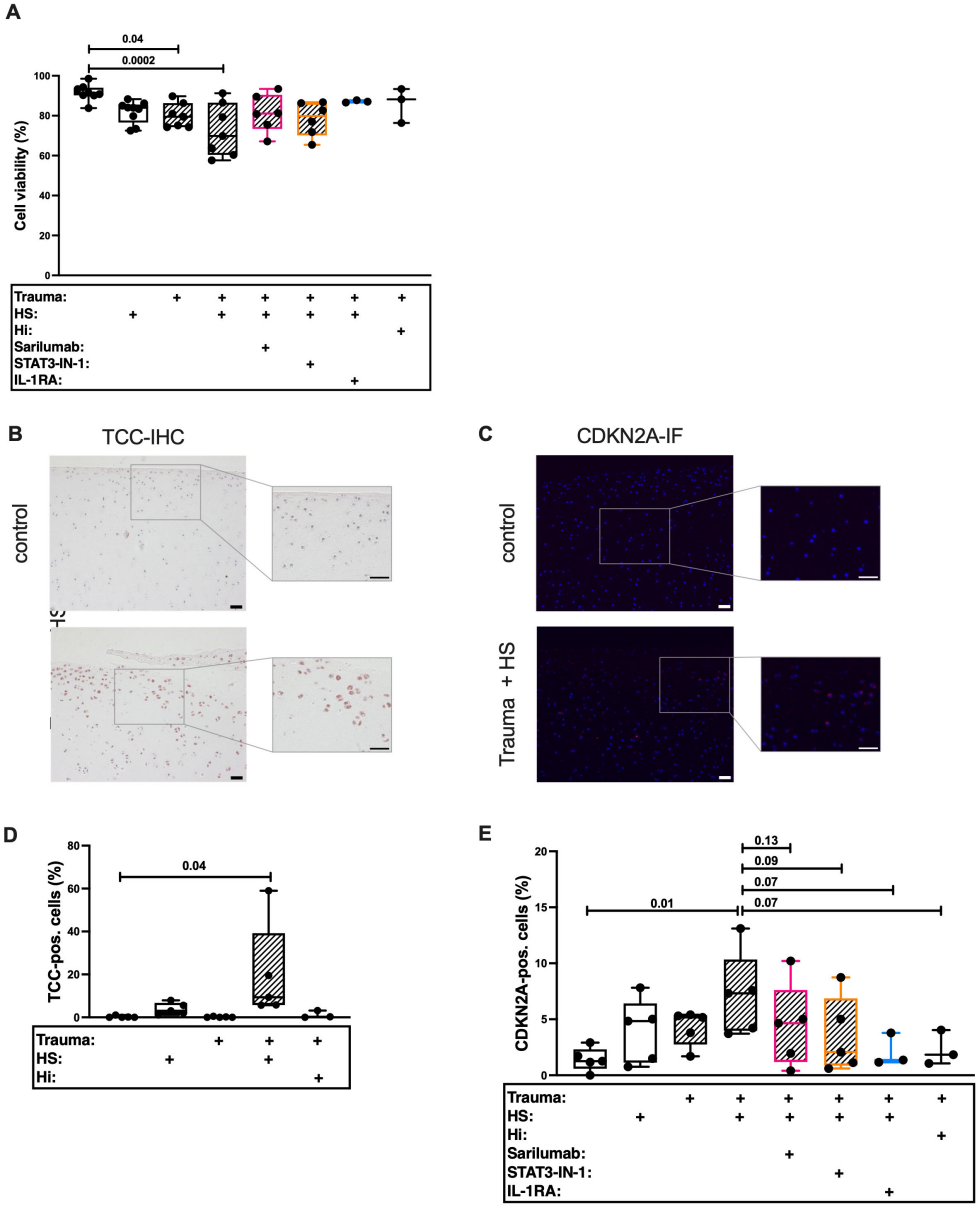
Histological evaluation of traumatized porcine cartilage exposed to 30% human serum (HS). Cartilage explants were cultured for 4 days. The first 24 h in presence of 30% HS. The following 3 days in presence of either 5 μg/mL Sarilumab, 5 μM STAT3-IN-1, or 50 ng/mL IL-1RA. **(A)** Cell viability assessed by Live/Dead staining of explants; n ≥ 3. Exemplary images of **(B)** TCC-IHC and **(C)** CDKN2A-IF. Scale bars equal 50 μm. Quantification of **(D)** TCC-IHC (n ≥ 3) and **(E)** CDKN2A-IF (n ≥ 3). Statistical analysis: one-way ANOVA with **(A, D)** Šidák´s correction, or with **(E)** Holm-Šidák´s correction.

In our last experiment, we investigated the underlying mechanism of TCC-induced SIPS in more detail. In this case we used porcine cartilage to study the effects of HS on healthy tissue. The porcine knee anatomy is similar to the anatomy of the human knee (44). In accordance to our human model (18), cartilage trauma and HS exposition had additive effects on cell death in porcine cartilage (**Figure 6A**) and was accompanied by enhanced TCC deposition (**Figures 6B, D**). In line with this, HS exposure and trauma had additive effects on the percentage of CDKN2A-positive cells (**Figures 6C, E**). We previously showed that HS exposition and enhanced trauma induced IL-6 secretion in human tissue (18). However, IL-6 alone did not induce the expressions of *CDKN1A* and *CDKN2A* (**Supplementary Figure 4**) in hAC. On the contrary, IL-6 induced the number of cells which were positively stained for the proliferation marker protein Ki-67 (**Supplementary Figure 5**). Furthermore, inhibition of IL-6 signaling via Sarilumab or STAT-IN-1 reduced CDKN2A expression by trend. This was also true when pro-inflammatory effects of IL-1β were inhibited by IL-1RA.

## 4 Discussion

Previous research indicates that the complement system is activated after cartilage trauma and might be involved in consequent OA progression. Accordingly, there is preliminary evidence that sublytic TCC influences the cell fate of surviving chondrocytes, including regulated cell death and phenotypical alteration (7–9, 18). However, the underlying mechanisms have not yet been completely clarified. With the present study, we wanted to elucidate if TCC deposition contributes to SIPS in the context of age-related and injury-induced OA. Furthermore, we investigated if SIPS is primarily induced by oxidative stress or by pro-inflammatory mediators, as both have been discussed as drivers of chondrosenescence. We demonstrated that expression of senescence-associated markers was elevated in the femoral condyles of aged CD59-ko mice as compared to WT mice. In line with this, CD59-ko mice developed more severe OA. By means of an *ex vivo* cartilage trauma model, we observed an additive effect of trauma and serum exposition regarding the development of a senescent phenotype in human and porcine chondrocytes. The expression of senescence markers was only reduced by specific inhibition of TCC formation but not by administration of the antioxidant NAC. Experiments with porcine cartilage showed that the inhibition of IL-6 or IL-1β signaling attenuated a senescent phenotype of chondrocytes after trauma and HS exposition. Taken together, these findings indicate a crucial role of sublytic TCC deposition and consequent SIPS in OA.

Originally, the TCC was described as a pore-forming complex which sole function was the elimination of foreign cells via osmolysis – an efficient strategy against gram negative bacteria or parasites (16). However, nucleated cells such as somatic cells have multiple defense mechanism (e.g., expression of CD59) to resist TCC-mediated osmolysis (45, 46). Residual TCC deposition on the cell membrane is not sufficient to directly kill the cell. However, due to the pore-forming character of TCC, a calcium influx can be observed upon sublytic TCC deposition (47, 48). In macrophages, TCC-mediated increase of intracellular Ca2+ was associated with a reduced mitochondrial membrane potential, which resulted in mitochondrial dysfunction and enhanced ROS production (17). Although, consequent oxidative stress promoted NLRP3 inflammasome activation (17), ROS accumulation is also considered as a crucial inducer of SIPS (29). However, the antioxidant NAC did not prevent the expression of senescence-associated genes after HS exposition and traumatization.

Furthermore, it was demonstrated that sublytic TCC induced IL-8 secretion and *IL6* expression in human endothelial cells, presumably mediated by NF-κB (49, 50). Accordingly, we previously reported that TCC deposition was associated with the expression of pro-inflammatory mediators, including *IL8* and IL-6 after traumatization and HS exposition in our human *ex vivo* model (18). Additionally, secretion of IL-1β was described upon TCC-mediated potassium efflux and consequent inflammasome assembly (16). All three proinflammatory cytokines are known SASP factors released by senescent hAC (25, 26). They can alter the phenotype of cells in a paracrine and autocrine manner, thus inducing senescence of adjacent cells (51). In the present study, we showed that specific TCC inhibition by CLU reduced *IL6* expression as well as the expression of other senescence-associated genes. Furthermore, *Il6* was upregulated in aged CD59-ko mice and correlated with age-related OA development. Although IL-6 alone was not sufficient to induce senescence in hAC, inhibition of IL-6 signaling by targeting either the IL-6 receptor using Sarilumab or the transcription factor STAT3 [pro-inflammatory signaling pathway of IL-6 (52)] by STAT3-IN-1 attenuated CDKN2A expression after trauma and HS exposition. In accordance with our data, the role of IL-6 in cartilage degeneration and regeneration is currently controversially discussed (53–55). Besides promotion of paracrine senescence, SASP factors, and in particular IL-6, are thought to mediate pro-regenerative processes, comprising proliferation, migration, and immunomodulation (56). In line with this, we recently reported cell protective and pro-mitotic effects by growth differentiation factor 15, a stress-responsive, pleiotropic cytokine and known SASP factor, in the context of PTOA (57).

Similar to IL-6, IL-1β did not directly induce senescence in hAC (58), even though inhibition of the IL-1β receptor reduced TCC-induced CDKN2A expression after trauma. We assume that multiple stimuli are needed simultaneously to orchestrate senescence in hAC, e.g., oxidative stress, pro-mitotic stimuli, and pro-inflammatory signaling. The latter might not result from a single mediator but rather a “cytokine cocktail”, in which IL-6 and IL-1β might represent important players.

Although we previously reported that cartilage trauma and blood exposure results in complement activation and subsequent TCC deposition on chondrocytes (8, 9, 18), the current findings of the age-related OA model imply that a preceding joint injury, is not mandatory. Thus, we presume that the local expression of complement components by joint tissues (e.g., the synovium) and a non-traumatic complement activation is involved in the development of age-related OA. As reported by Cheng et al., 2022, synovial C3 levels, but not that of blood plasma, were positively associated with primary OA severity (59). It is known that chondrocytes and synovial cells express complement components. Thus, respective activation products, such as C3a-desArg, soluble TCC, C4d, and C3bBbP, were also found in the synovial fluid of healthy individuals (12, 13). This indicates a low basal activation of the complement system in the knee joint. In humans, the complement system is often dysregulated in age and an uncontrolled activation has been described (60, 61). Similarly, the CD59-def mice used in the present study lack an important TCC regulatory mechanism. Although the presence of the TCC could not be determined, it can be assumed that the cells of aged CD59-ko animals are more prone to TCC deposition, as we confirmed by siRNA-mediated knockdown of CD59 in hAC. In the aging mouse model, we found higher levels of senescence-associated markers in femur condyles of CD59-ko mice as compared to that of WT mice, implying a potential association between susceptibility to TCC deposition and an accumulation of senescent and therefore dysfunctional chondrocytes. Summing up, the findings of our mouse model indicate that TCC-induced SIPS is a possible pathomechanism of age-related OA.

Previous studies indicated that the complement system is activated after joint injuries (13) due to an enhanced protease activity and the accumulation of DAMPs and ROS (15, 62, 63). Furthermore, inflammatory cytokines, found after acute knee injury (64), induce the expression of complement components and thus amplify complement activation (65, 66). Additionally, intraarticular bleeding, often observed after knee injuries (67), might contribute to complement activation in the joint (11). Our present findings reaffirmed that tissue injury and HS exposition have additive effect on the expression of CDKN1A and CDKN2A in chondrocytes. In conclusion, sublytic TCC and consequent induction of chondrosenescence might play a role in PTOA progression.

Several studies demonstrated that an injury-related hemarthrosis has harmful effects on cartilage homeostasis (9, 68). The assumed pathophysiology of blood-induced cartilage degeneration includes exposure to iron, expression of pro-inflammatory cytokines and MMPs (68), as well as complement activation and subsequent TCC deposition (9). Taken our results into account, we believe that sublytic TCC deposition and consequent induction of a senescent phenotype are crucially involved in the disruption of the cartilage integrity. Over all, a rather short exposition to HS (24 h or 4 days) was sufficient to induce a senescent phenotype in healthy porcine tissue. Future studies have to explore whether the observed senescence is transient or stable in the long term. Nevertheless, an immediate treatment after joint injury is advisable to diminish the harmful effects of TCC deposition. The main focus should be the prevention of the TCC assembly, either on the level of the TCC or more upstream in the complement cascade.

Previous studies showed that TCC deposition is associated with various, mainly age-associated diseases. Often, the TCC is not the main cause, but is involved in the pathogenesis. For example, TCC deposition was found on dystrophic neurites in the brain of patients with Alzheimer’s disease (69). Furthermore, affected tissues were positive for TCC in patients with either Parkinson’s disease (70) or age-related macular degeneration (71, 72). Additionally, enhanced soluble TCC levels were detected in the plasma of amyotrophic lateral sclerosis (73) and schizophrenia patients (74). In the context of musculoskeletal diseases, it has been reported that TCC deposition is associated with intervertebral disc degeneration (75–77). Some studies also evaluated whether presence of TCC is associated with an altered phenotype of the targeted cells. Zeng et al. reported that sublytic TCC deposition enhanced the expression of catabolic enzymes and inflammatory markers in chorioretinal endothelial cells (78). Similar observations were found in annulus fibrosus cells which were stimulated with zymosan to induce TCC deposition (75). Overall, consequences of sublytic TCC deposition could not only be involved in (PT)OA pathogenesis but also in other diseases.

One limitation of the present study is that only male mice were included in the *in vivo* experiment. This was chosen due to the fact that male mice are more prone to develop age-related OA (36). Furthermore, female mice of some breeding strains have lower serum concentrations of C6 and C9 (37). Using female mice in a follow-up study might be advisable to determine whether reduced C6 and C9 levels, and thus reduced TCC deposition, might be an explanation for the lower OA severity observed in aged female mice. Another reason to include female mice is that women have in general an enhanced risk to develop OA.

Overall, we provided first evidence that complement activation and consequent TCC deposition is associated with a SIPS-like phenotype in age-related and trauma-induced OA. We suggest that chondrosenescence is more likely induced by sublytic TCC deposition and subsequent cytokine release rather than oxidative stress. A local inhibition of the complement system, particularly targeting TCC formation, might be a promising therapeutic approach to reduce the detrimental effects of TCC, comprising cell death, inflammation, and SIPS.

## Data Availability

The original contributions presented in the study are included in the article/**Supplementary Material** material, further inquiries can be directed to the corresponding author.
