# Supplementary material for: Terminal complement complex deposition on chondrocytes promotes premature senescence in age- and trauma-related osteoarthritis
Source: Front Immunol. 2025 Jan 14;15:1470907. doi: 10.3389/fimmu.2024.1470907 (PMC11772281; doi:10.3389/fimmu.2024.1470907)
Supplement: Supplementary file 1 [file DataSheet1.pdf]

Supplementary Material

Supplementary Table 1. Weight of mice at day of sacrifice

	Age [weeks]	Weight [g]	Standard deviation
C57BL/6 wildtype	72	34.6	5.16
CD59-knockout	13	28.8	0.96
	72	39.7	4.80
C6-deficient	13	24.7	0.52
	72	31.3	2.12

Supplementary Table 2. Used primers and TagMan Gene Expression Assays

Gene	Species	Assay ID / primer sequence
CD59	human	Hs000174141_m1
CDKN1A	human	Hs00355782_m1
CDKN2A	human	Hs00923894_m1
GAPDH	human	Hs02758991_g1
HPRT1	human	Hs02800695_m1
IL6	human	Hs00174131_m1
SOD2	human	Hs00167309_m1
Cdkn1a	murine	forward: (5´-CCT CCC AAG ATA GCC GAG TT-3´)<br>reverse: (5´-AGA CGA CAC AGG TGA GGA AG-3´)
Cdkn2a	murine	Hs00494449_m1
Gapdh	murine	Mm99999915_g1
Hprt1	murine	Mm03024075_m1
Il1b	murine	forward: (5´-ACA AGG AGA ACC AAG CAA CG-3´)<br>reverse: (5´-GGG TGT GCC GTC TTT CAT TA-3´)
Il6	murine	Mm00446190_m1
Mmp13	murine	Mm00439491_m1
Tp53	murine	forward: (5´-GGA AAT TTG TAT CCC GAG TAT CTG-3´)<br>reverse: (5´-GTC TTC CAG TGT GAT GAT GGT AA-3´)

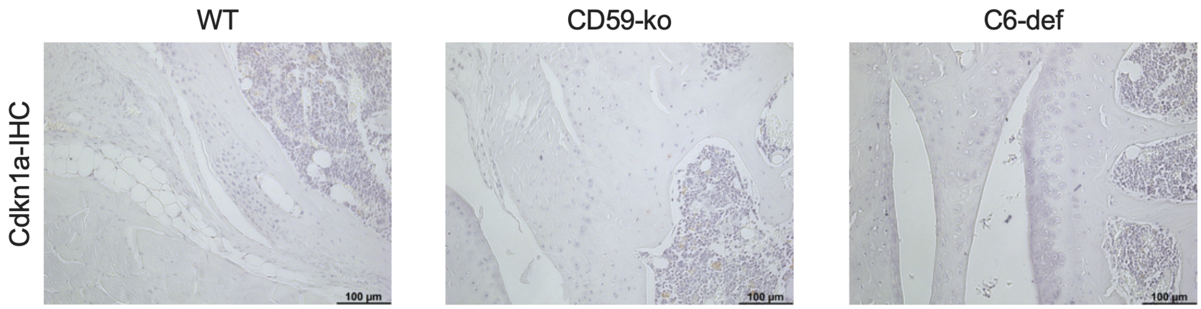

Supplementary Figure 1. Representative images of Cdkn1a-IHC of 72-weeks-old WT, CD59-ko, and C6-def mice. Scale bars equal 100 µm. Positive cells were only found in the bone narrow and growth plate (not shown).

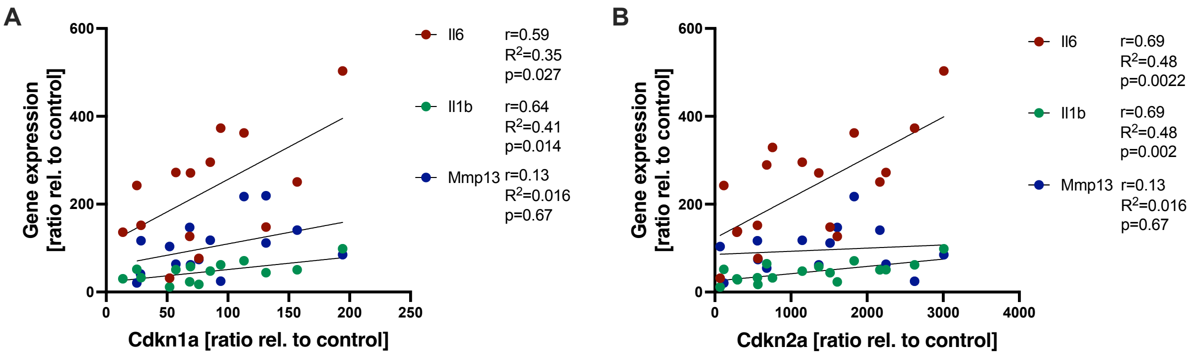

Supplementary Figure 2. Pearson correlation of *Cdkn1a* (A) and *Cdkn2a* (B) expression with the expression of the SASP factors *Il6*, *Il1b*, and *Mmp13*. 72-weeks-old WT, CD59-ko, and C6-def mice; n ≥ 14.

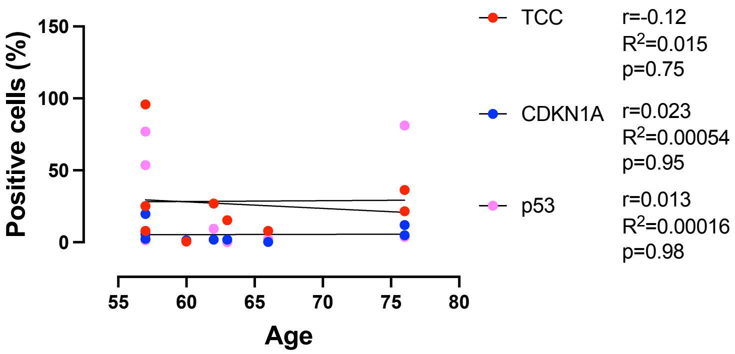

Supplementary Figure 3. Pearson correlation of the age of the donor and the percentage of TCC-, CDKN1A-, and p53-positive cells. n ≥ 8.

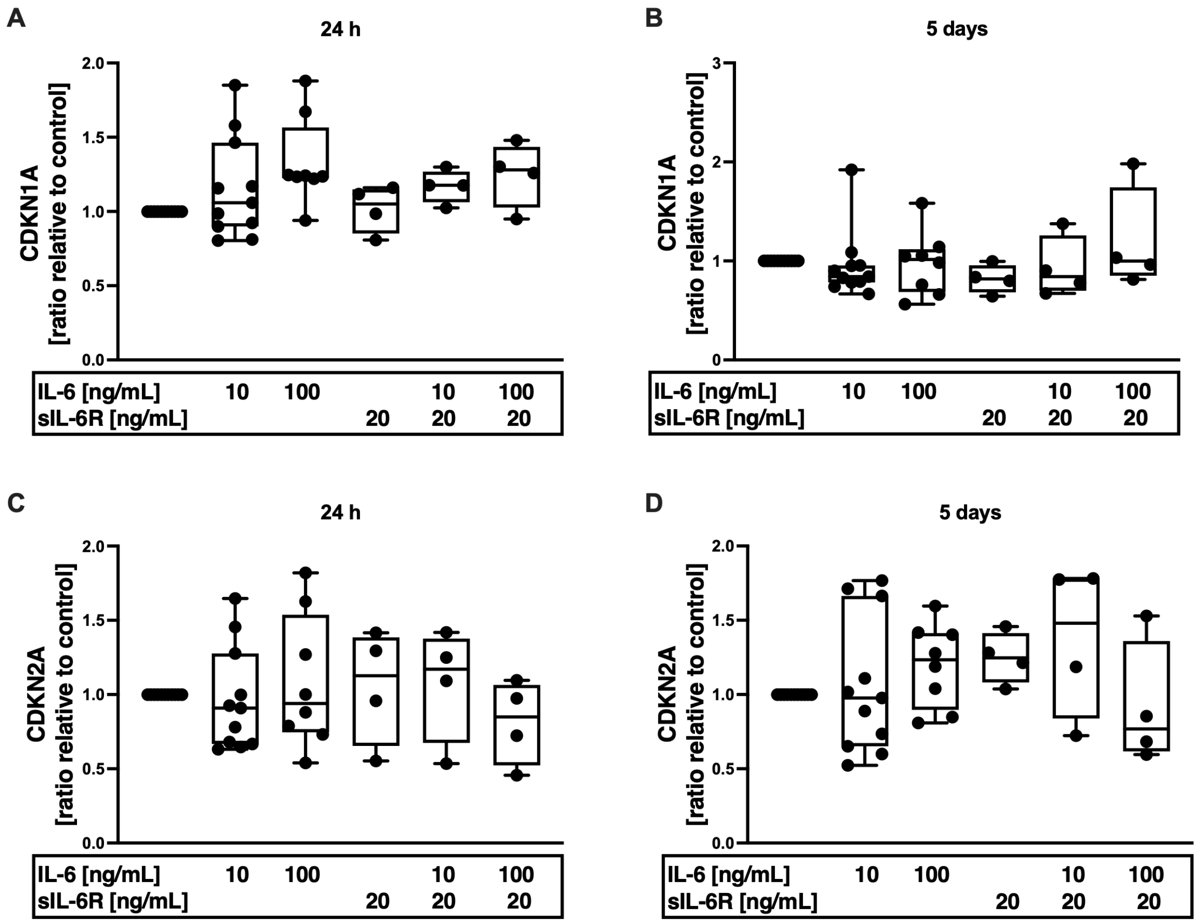

Supplementary Figure 4. Gene expression of hAC treated with IL-6 w/ and w/o soluble IL-6 receptor (sIL-6R). mRNA levels of **(A)** CDKN1A and **(B)** CDKN2A was analyzed in hAC stimulated for 24 h or 5 days with 10 or 100 ng/mL IL-6 in presence and absence of 20 ng/mL sIL-6R. Normalized to untreated hAC; n ≥ 4.

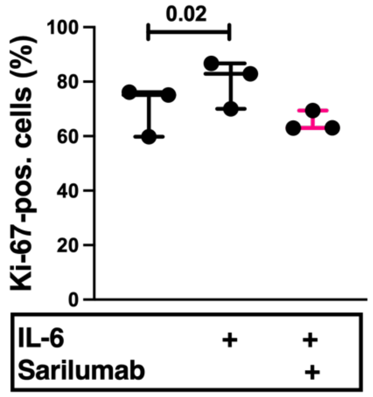

Supplementary Figure 5. Ki-67 staining of hAC treated for 48 h with 100 ng/mL IL-6 in presence and absence of 5 µg/mL Sarilumab. The number of Ki-67 positive cells was evaluated by means of an immunofluorescence staining. Cells were permeabilized with 0.1% TritonX100 (Merck) prior to the incubation with the Ki-67 antibody (1:250, ab16667, abcam); n = 3. Statistical analysis: one-way ANOVA with Tukey correction.
